# Structural Parameters Optimization of Elastic Cell in a Near-Bit Drilling Engineering Parameters Measurement Sub

**DOI:** 10.3390/s19153343

**Published:** 2019-07-30

**Authors:** Long Zhao, Yifei Yan, Xiangzhen Yan, Lei Zhao

**Affiliations:** 1 College of Mechanical and Electronic Engineering, China University of Petroleum, Qingdao 266580, China; 2 Qil & Gas CAE Technology Research Center, China University of Petroleum, Qingdao 266580, China; 3 College of Pipeline and Civil Engineering, China University of Petroleum, Qingdao 266580, China

**Keywords:** near-bit drilling engineering parameters measurement sub, elastic cell, structural parameters optimization, NSGA-II, transient dynamic analysis, systematic method framework

## Abstract

The downhole engineering parameters measurement sub is a key component of the rotary steerable drilling system. To enable a measurement sub to serve reliably under downhole complex conditions, the structural parameters optimization of its key but weak elastic cell is systematically studied. First, the multiple relations among measurement sensitivities, structural stiffnesses, and strength during structural parameters design are summarized. Second, the selection of the structural parameters of the elastic cell is characterized as a multi-objective optimization model, which is solved using the non-dominated sorting genetic algorithm II (NSGA-II). Furthermore, the finite element method (FEM) is used to verify the measurement performance and static strength of the proposed structure. Finally, transient dynamics analysis is applied to investigate the dynamic strength of the designed structure. The results show that the proposed parameters optimization strategy can quickly obtain the database for the structural parameters design of an elastic cell. The static analysis results based on the FEM further verify the effectiveness of the proposed method. Transient dynamic analysis also reveals the relative rigor of the proposed methodology framework to some extent. This work has practical significance for improving the drilling efficiency and reducing drilling risks. In addition, this proposed methodology has good extensibility.

## 1. Introduction

With the consumption of conventional oil and gas resources, some unconventional resources, such as shale gas, tight oil and gas, are becoming popular [1]. For the development of these unconventional resources, horizontal wells, multi-lateral wells, and extended reach wells are usually required [2]. The use of complex wells usually poses challenges to drilling techniques [3]. Further, drilling is a high-cost and high-risk operation [4,5]. Therefore, drilling optimization is an important issue. Driven by this problem, Rotary steerable drilling technology was developed [6]. One of the core elements of this technology is the real-time and accurate measurement of near-bit drilling engineering parameters [7]. By properly designing or arranging the sensors on the near-bit measurement sub, multiple drilling engineering parameters, such as weight on bit (WOB), torque, and bending moment, can be accurately measured. After the measured parameters are transmitted, interpreted, and analyzed, they can be converted into driving instructions for the relevant equipment, and then the optimal control of the drilling process is realized. Therefore, the study of drilling parameters measurement is an important issue in the research process of advanced drilling technology [8].

Ma and Chen [8] designed a drilling engineering parameters measurement system for use in deep wells and systematically described the design parameters, calibration, and field test results of the developed sensor. The instruments can meet the requirements for rotary steerable drilling and conventional drilling. Liu et al. [9] developed a new type of drilling parameters measurement instrument and corresponding parameters processing model. This instrument can measure WOB, torque, and lateral force simultaneously. To ensure the reliability of the navigation system during drilling operation, Seyed Moosavi [10] designed a 4-sensor (accelerometers) structure for a Measurement While Drilling (MWD) tool. This tool can perform an autocalibration process, diagnose faults, and isolate faulty accelerometers. Li et al. [11] designed an experimental rig that combines near-bit force measurement and conducted a drill string acoustic transmission study. The designed force sensor device can simultaneously detect several near-bit parameters applied on the simulated drill string, and the developed acoustic transmission device is suitable for transmitting the measured force data along the simulated drill string via elastic waves. Hu et al. [12] proposed a kind of design scheme for a downhole engineering parameters measurement system while drilling, which covers the hardware level and software level, and the function and reliability of the system have been verified by the in-site test. To investigate the mechanical responses of downhole tools in highly-deviated waterflooding wells, Liu et al. [13] developed a downhole parameters measurement tool for measuring axial force, annular pressure, and temperature. This study discussed the calibration of the measurement instrument to ensure its reliability and feasibility. Wang et al. [14] discussed the effects of temperature variations and well pressure differentials on the accuracy of WOB measurement sensors. They discussed the shortcomings of the conventional temperature compensatory method and WOB correction method and proposed two new methods. Xu et al. [15] pointed out that there is a large difference between the measured values and the real values in current WOB measurements. This study conducted a force analysis of measuring tools, an axial force calibration and a correction experiment and then proposed a calculation method of the actual WOB by correcting the measured data.

Previous studies have contributed much to the research in the field of drilling parameters measurement. However, the main concern in the above studies is sensor signal postprocessing or the transmission process of a measurement system. To date, little attention has been paid to the front of a measuring system (i.e., the elastic cell). The sensitivity and accuracy of a sensor are closely related to its elastic cell. An elastic cell is, therefore, one of the key components of a sensor [16]. The reasonable design of an elastic cell for direct response to physical signals is the premise of the reliable operation of a downhole parameters measurement system. As mentioned by Wang et al. [17] and Zhang [18], the measurement noise caused by the vibration of the drill collar and the electromagnetic waves of logging while drilling is very large during the measurement of the near-bit engineering parameters. Geng et al. [19] further pointed out that the mechanical magnification (i.e., the mechanical deformation) of a sensing cell should be increased as much as possible, rather than relying on electrical magnification, because this can improve the anti-interference performance of a measurement system. Therefore, it is necessary to conduct a detailed study of the elastic cell of a measurement tool. Sensitivity and stiffness are two basic characteristics of an elastic cell of a measurement sub. [20]. However, there is always a contradiction between sensitivity and stiffness in the design of an elastic cell [21]. In other words, if the sensor is expected to have a higher sensitivity, the stiffness of its elastic cell can be lower. The static characteristics and dynamic characteristics of a sensor depend to a large extent on the stiffness of the elastic cell. Thus, an elastic cell is also expected to have a higher stiffness [22]. Meanwhile, it should be noted that the downhole environment is very complex. The possibility of failure for downhole tools is high, and the corresponding failure consequences are usually severe [23,24]. The elastic cell used to capture physical deformations is often designed as a structure with weakened strength. This means that the structural integrity of the elastic cell must be considered when designing a sensor for downhole environments. Therefore, the design of the sensor elastic cell of a downhole engineering parameters measurement sub is a complex task. The sensitivity, stiffness, and strength of an elastic cell should be simultaneously taken into account in a design.

Although the elastic cell of a sensor is a key component to ensure the accuracy and reliability of the entire measurement system, as mentioned previously, the specific studies on elastic cells are still very limited. Fan et al. [25,26] discussed the optimization design of an elastic cell of a downhole engineering parameters measurement sub, and considered the trade-off between measurement sensitivity and stiffness in the design. This work made contributions to the design research of elastic cells. In their study, however, bending moment measurements were ignored, and a clear design framework of elastic cells was not proposed. This paper conducts a more specific study on the elastic cell in a downhole engineering parameters measurement sub based on previous studies. A systematic and clear methodology for the structural parameters optimization of elastic cells is proposed. This methodology is easy to use, expand, and connect with other studies on topics such as sensor calibration and data transmission. In the design of elastic cells, three measuring parameters are considered: WOB, torque and bending moment. It is worth noting that the measurement and acquisition of bending moments is very important because directional wells are being widely used. The incorporation of the non-dominated sorting genetic algorithm II (NSGA-II) into the proposed method framework is proposed. NSGA-II is an efficient and robust evolutionary multi-objective optimization algorithm and has been widely adopted in other fields. It is also proposed that dynamics analyses are incorporated into the optimization of elastic cells. A dynamics analysis can provide more useful information and is a potential means for improving designs.

This paper is organized as follows. Section 2 gives the proposed technical framework. Section 3 analyzes the relationship between the structural parameters and their sensitivities, stiffnesses, and strengths. The establishment and solution of the multi-objective optimization model are discussed in Section 4. Design verification and examination based on the FEM are carried out in Section 5, followed by the conclusions in Section 6.

## 2. Proposed Methodology Framework

**Figure 1:**
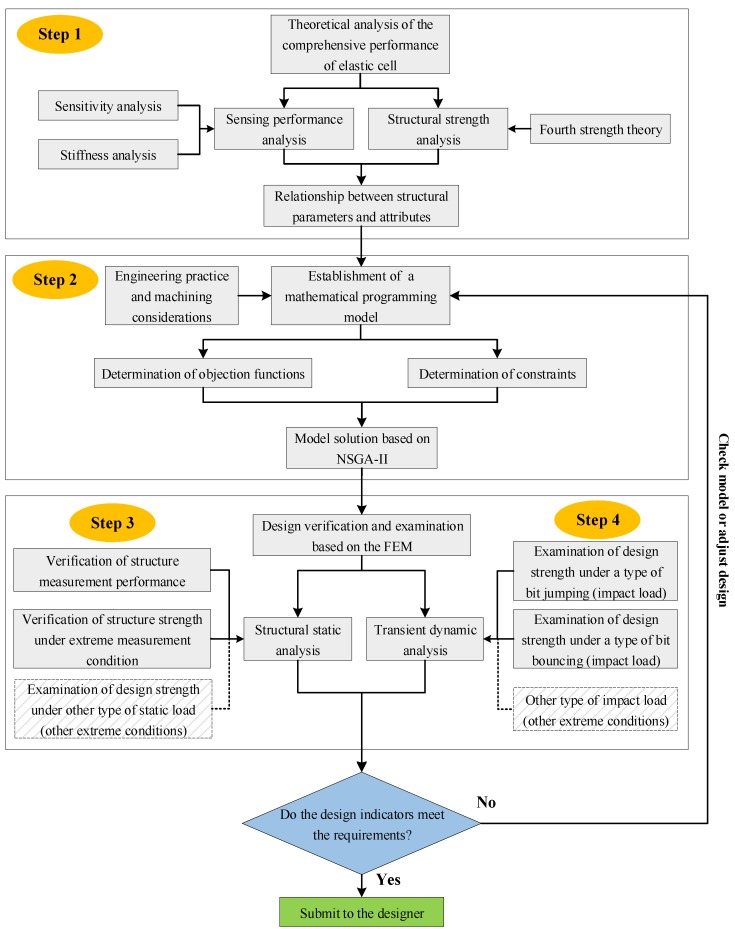
Proposed methodology framework for structural parameters optimization.

The methodology for optimizing the structural parameters of an elastic cell in a near-bit drilling engineering parameters measurement sub proposed in this paper is shown in Figure 1. The proposed methodology adheres to the following four steps.

1. Sensitivity, stiffness, and strength analysis of an elastic cell. In this step, the expressions of the sensitivities and stiffnesses of the elastic cell are derived based on the mechanics of materials. As a result, the relationships among the structural parameters of the elastic cell and each sensitivity and stiffness can be determined. Meanwhile, the stress state of the elastic cell is analyzed based on the mechanics of materials and elastic theory. Thus, the relationships among the structural parameters of the elastic cell and the structural strength can be determined. In conclusion, the relationships among the structural parameters of the elastic cell and its main performances are described systematically.
2. Establishment and solution of a mathematical programming model. A multi-objective optimization model is established to characterize the multiple relationships among measurement sensitivities, structural stiffnesses, and strength in the design of structural parameters. In the modeling process, the sensitivities and stiffnesses are specified as objective functions, and the strength indicators and some engineering or process requirements are specified as constraints. NSGA-II is used to solve the multi-objective optimization model. The optimization results can provide a flexible theoretical solution database.
3. Design verification and examination based on the finite element method (FEM). The measurement performance and static strength (in the extreme measurement condition) of the proposed design are verified by the static analysis. The analysis should be regarded as the validation of the optimization algorithm. If the error is large, return to step 2 and examine the objective functions or constraint functions in the optimization model. In addition, case-specific conditions can be set to examine the integrity of the proposed structure. This may provide feedback information on the proposed design. This part reflects the scalability of the proposed model.
4. Design examination based on transient dynamic analysis. Transient dynamic analysis is applied to examine dynamic responses of the proposed design under the impact loads of some extreme conditions. If the structure cannot bear the impact load under the working conditions studied, go back to step 2 and adjust the constraint functions in the optimization model. In this study, the transient dynamic models of the sub under two typical working conditions are established, and the dynamic response of the structure under the corresponding impact load is investigated. Similar to step 3, some case-specific conditions can be added to the dynamic analysis in specific applications.

## 3. Sensitivity, Stiffness and Strength Analysis of Elastic Cell

### 3.1. Basic Principle of Parameters Measurement

**Figure 2:**
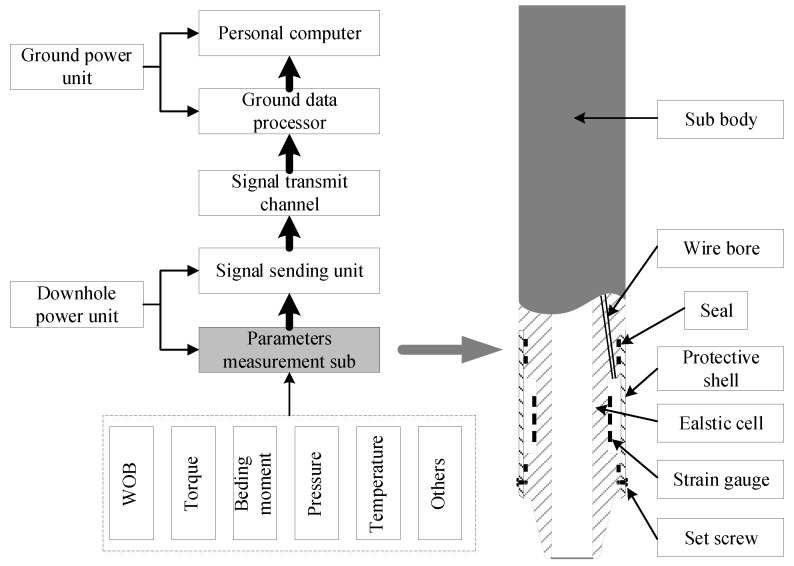
Schematic diagram general parameters measurement system and measurement sub.

Downhole engineering parameters usually include WOB, torque, bending moment, annulus pressure, pressure inside the drill collar, acceleration, and temperature. The measurement of the latter four parameters can directly rely on existing standard sensors. In this case, attention should only be paid to the installation and power supply of the sensors. However, in light of the particularity of the environment, the measurement of the first three parameters usually requires specially designed sensors [19]. Therefore, this study mainly analyzes the structural parameters optimization of the elastic cell in the measurement sub used to measure WOB, torque and bending moment. Figure 2 shows a schematic diagram of the general parameters measurement system and measurement sub [8,19,26,27].

For a measurement sub, the elastic cell and resistance strain gauges constitute the sensing part. The parameters acquisition principle of a measurement sub is that the measured physical quantities, such as WOB, torque, and bending moment, cause elastic deformation of the elastic cell, and the strain gauges attached to the surface of the elastic cell convert the mechanical responses of theses physical quantities into changes in the resistance values. In this way, the sensor converts the changes of physical quantities measured into changes of electrical signals [16].

To illustrate the proposed methodology, this paper takes the following design parameters as an example. The measuring range of the WOB is 0–300 kN, the measuring range of the torque is 0–8 kNm and the measuring range of the bending moment is 0–10 kNm. The maximum internal pressure of the drill string is 50 MPa, and the maximum annulus pressure is 35 MPa. The outer diameter of the measurement tool is Φ172 mm (the connection thread is NC50-67), and the applicable borehole size is Φ215.9 mm. In addition, the material of the sub is 42CrMo.

### 3.2. Sensitivity and Stiffness Analysis of Elastic Cell

Sensitivity and stiffness are two basic characteristics of an elastic cell. Sensitivity [26] refers to the ratio between the output strain and the input load of the elastic cell, as shown in Equation (1). The sensitivity should not be depicted by the ratio of a system’s terminal meter readings to the input load because such a value is related to the electrical magnification of the instrument, which does not reflect the sensitivity of the elastic cell itself. Stiffness [22] refers to the external load required to cause the elastic cell to produce the unit deformation and can be characterized by Equation (2). Stiffness directly determines the static characteristics (e.g., sensitivity, linearity, and hysteresis) and dynamic characteristics (e.g., natural frequency, frequency response, and transient response) of a sensor. Obviously, stiffness is an important performance index of a sensor:

(1) S=ε/F,

where *S* is the sensitivity of the elastic cell, *F* is the measured load, and *ε* is the strain at the sensing part under a corresponding load:

(2) K=F/ΔL,

where *K* is the stiffness of the elastic cell and ∆*L* is the deformation at the sensing part under a corresponding load.

**Figure 3:**
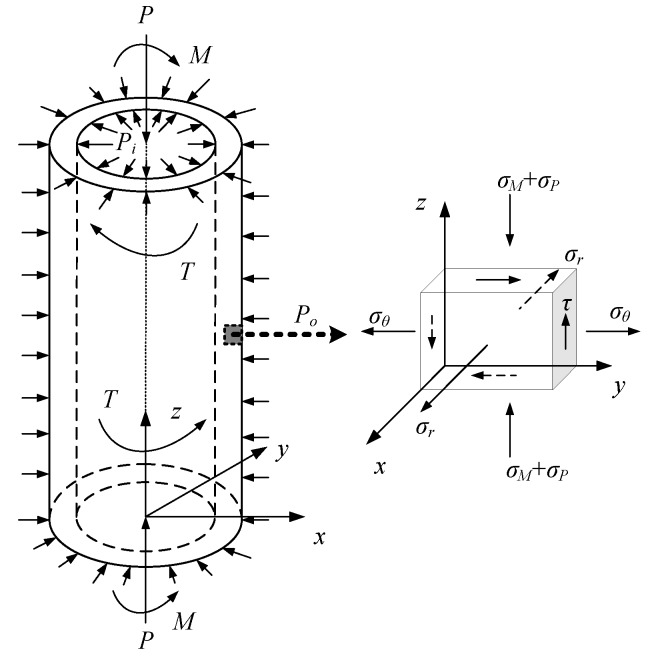
Load conditions of a measurement sub.

Structurally, a measurement sub can be considered as a thick-walled cylinder, and its load status is shown in Figure 3. The load conditions of a measurement sub working in a downhole environment are complex, so simplifications are made here. WOB *P*, driving torque *T*, bending moment *M,* which can be decomposed into *M_x_* and *M_y_*, annulus pressure *P_o_* and inner pressure *P_i_* of a drill collar are mainly considered. Note that the outer protective shell of strain gauges is usually fixed on the sub body by means of set screws, and only bears the bending moment and annulus pressure. According to Equations (1) and (2) and the mechanics of materials [28], the expressions of the sensitivities and stiffnesses of the elastic cell can be derived, as shown in Table 1.

As seen from Table 1, the structural parameters that determine the sensitivities and stiffnesses are *d*, *t*, *L*, *d_o_*, and *t_o_*. Engineering considerations require that the outer diameter of the outer protective shell of strain gauges should be the same as that of the sub body. This consideration is essentially based on the continuity requirements of a structure. If the outer protective shell protrudes from the entire sub, it is prone to wear during drilling or tripping. Then, the parameters *d_o_* and *t_o_* can be considered as a pair of dependent variables for the case in which the outer diameter of the protective shell has been determined. In other words, only the parameter *t_o_* needs to be specified in a design. A protective shell is only used to protect strain gauges from the external environment. Therefore, it only needs to ensure the strength of a protective shell, and the sealing problem is not discussed here. Moreover, the load status of a protective shell is relatively simple. This means that ensuring its structural integrity is not difficult. Thus, *t_o_* can be selected according to relevant experience or studies [25,29,30,31]. The focus of this paper is on the optimization of the structural parameters of elastic cells, so no more discussion is made on the parameter *t_o_*. As long as a value of *t_o_* is given, the proposed optimization method can usually provide the corresponding optimal structural parameters of the elastic cell. If the optimization process does not converge or the satisfactory solutions cannot be obtained, the value of *t_o_* shall be adjusted. In this work, let *t_o_* = 10 mm (i.e., *d_o_* = 152 mm) according to experience.

**Figure 4:**
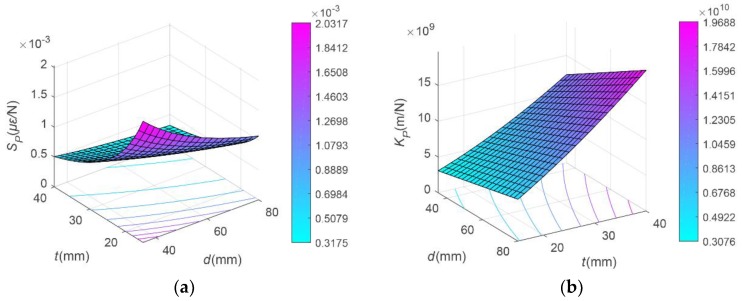

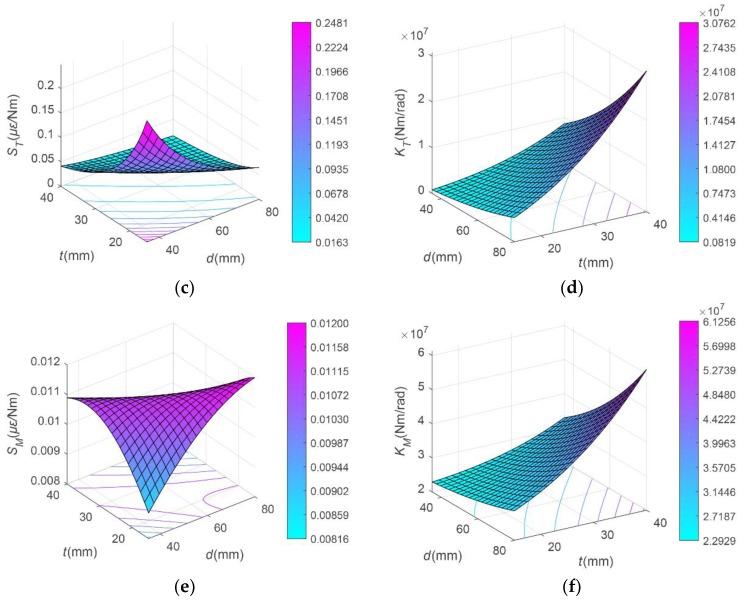
Relationships among *d*, *t*, and sensitivities and stiffnesses: (**a**) *S_P_*; (**b**) *K_P_*; (**c**) *S_T_*; (**d**) *K_T_*; (**e**) *S_M_*; (**f**) *K_M_*.

Figure 4 shows the relationships among *d*, *t*, and sensitivity and stiffness of each measurement, when *L* = 160 mm. Note that the value of *L* is assigned according to experience [19]. Meanwhile, the parameters *d* and *t* take values in the appropriate interval. These parameters will be discussed later. As shown in Figure 4a–d, as *d* and *t* increase, *S_P_* and *S_T_* decrease, while *K_P_* and *K_T_* increase. This reflects the contrariety between sensitivity and stiffness. The bending moment parameters have a relatively special relationship with *d* and *t*. When a bending moment acts, the elastic cell is carried together with its outer protective shell. As a result, the structural superposition component is contained in the expressions of *S_M_* and *K_M_*. Figure 4e,f show the relationships among *d*, *t*, and *S_M_*, *K_M_*. It is worth noting that *S_M_* first increases and then decreases with the increase of *d* and *t*. In conclusion, the sensitivity and corresponding stiffness of each measurement (i.e., WOB, torque or bending moment) are irreconcilable or inconsistent in the assignment process of *d* and *t*. In addition, *L* is only related to the stiffness of each direction and is negatively correlated with it.

### 3.3. Structural Strength Analysis of Elastic Cell

As the section of the structure that is thinned, the elastic cell is the most dangerous zone of the entire short section. Therefore, the selection of the structural parameters of an elastic cell must consider not only the basic characteristics of the sensor but also structural integrity. As previously mentioned, the structure of an elastic cell can be considered as a thick-walled cylindrical structure. Figure 3 shows the stress state of the unit body in the elastic cell. The stresses on the unit body include the axial stress $\sigma_{P}$ caused by the WOB *P*, the axial stress $\sigma_{M}$ caused by the bending moment *M*, the shear stress *τ* caused by the driving torque *T*, the radial stress $\sigma_{r}$, and the circumferential stress $\sigma_{\theta }$ caused by the inner pressure *P_i_*, and the outer pressure *P_o_* of the drill collar. The analytic formulae of these stresses can be derived based on the mechanics of materials and elastic theory [28,31,32].

The axial stress caused by *P* is

(3) $$\sigma_{P}=\frac{4P}{\pi \left[{\left(d+2t\right)}^{2}-d^{2}\right]}.$$

The axial stress caused by *M* can be expressed as:

(4) $$\sigma_{M}=\frac{64M\rho }{\pi \left[{\left(d+2t\right)}^{4}-d^{4}+{\left(d_{o}+2t_{o}\right)}^{4}-d_{o}{}^{4}\right]}.$$

Thus, the synthetic axial stress is

(5) $$\sigma_{z}=\sigma_{P}+\sigma_{M}=\frac{4P}{\pi \left[{\left(d+2t\right)}^{2}-d^{2}\right]}+\frac{64M\rho }{\pi \left[{\left(d+2t\right)}^{4}-d^{4}+{\left(d_{o}+2t_{o}\right)}^{4}-d_{o}{}^{4}\right]}.$$

The shear stress caused by *T* is

(6) $$\tau =\frac{32T\rho }{\pi \left[{\left(d+2t\right)}^{4}-d^{4}\right]}$$

where *ρ* is the distance from any point on the section to the center of the circle.

The radial stress and circumferential stress of the structure under *P_i_* and *P_o_* are obtained by the Lame formula:

(7) $$\sigma_{r}=\frac{P_{i}d^{2}-P_{o}{\left(d+2t\right)}^{2}}{{\left(d+2t\right)}^{2}-d^{2}}-\frac{d^{2}{\left(d+2t\right)}^{2}\left(P_{i}-P_{o}\right)}{4\left[{\left(d+2t\right)}^{2}-d^{2}\right]\rho^{2}},$$

(8) $$\sigma_{\theta }=\frac{P_{i}d^{2}-P_{o}{\left(d+2t\right)}^{2}}{{\left(d+2t\right)}^{2}-d^{2}}+\frac{d^{2}{\left(d+2t\right)}^{2}\left(P_{i}-P_{o}\right)}{4\left[{\left(d+2t\right)}^{2}-d^{2}\right]\rho^{2}}.$$

The unit surface with zero shear stress is one of the principal planes, and the normal stress on the principal plane is a principal stress. Thus, $\sigma_{r}$ is a principal stress. The other two principal stresses can be determined according to the plane stress state:

(9) $$\begin{array}{c} \sigma_{1}\\ \sigma_{3} \end{array}\}=\frac{1}{2}\left(\sigma_{\theta }+\sigma_{z}\right)\pm \sqrt{{\left(\frac{\sigma_{\theta }-\sigma_{z}}{2}\right)}^{2}+\tau^{2}}.$$

By substituting Equations (7) and (9) into the fourth strength theory, the equivalent stress of the structure can be derived:

(10) $$\sigma_{e}=\frac{\sqrt{{\left(\sigma_{z}-\sigma_{\theta }\right)}^{2}+{\left(\sigma_{\theta }-\sigma_{r}\right)}^{2}+{\left(\sigma_{r}-\sigma_{z}\right)}^{2}+6\tau^{2}}}{\sqrt{2}}.$$

Obviously, the structural parameters *d* and *t*, which affect the sensing performance of the elastic cell, are also related to its strength. This means that the selection of the size parameters of an elastic cell should simultaneously take into account the safety of the structure, the sensitivity of each measurement, and its stiffness. Therefore, although the elastic cell has a simple structure, the load status is complicated, and there are multiple indexes that must be satisfied. In the conventional elastic cell design, the structural size parameters are usually determined by a trial and error method. This method is characterized by repeated, varied attempts until the desired goal is met. Obviously, such a process is often a complex and time-consuming task. To improve design efficiency, a mathematical programming model is proposed in this work.

## 4. Establishment and Solution of a Mathematical Programming Model

### 4.1. Establishment of the a Mathematical Programming Model

#### 4.1.1. Initial Consideration of the Objective Function

Due to the particularity of the service environment, the wall thickness of an elastic cell in downhole parameters measurement sub is usually thicker. Therefore, when the electromotive strain method is used for parameters measurement, the output signal of the bridge circuit is relatively weak [17,18]. Moreover, to improve the anti-noise performance of the system, the requirement for electrical amplification should be reduced [19]. This means that the mechanical deformation of an elastic cell should be increased as much as possible to increase the sensitivity. High stiffness is also important for the elastic cell of a measurement sub [20,26]. As mentioned above, high stiffness allows an elastic cell to have good sensing properties including static and dynamic properties [22,33]. Moreover, because the measurement sub works near the bit, it will withstand various complex loads such as shocks and vibrations. To improve the measurement reliability, the elastic displacement of the elastic cell under a load should be reduced as far as possible. This also requires the elastic cell to have high stiffness [16]. As discussed in Section 3.2, there is a contradiction between the acquisition of the sensitivity and stiffness when designing an elastic cell. This means that a comprehensive consideration between the two design indices is necessary in a design. Therefore, in principle, the sensitivity and stiffness of each measurement should be regarded as the objective function of the optimization model.

#### 4.1.2. Calculation of Sensitivity Boundary

To ensure the reliability of the sensitivity design and its compatibility with the signal postprocessing system, the value of the sensitivity parameters of the elastic cell should be considered as a whole [26]. This work assumes that some performance parameters (e.g., the measurement range) of the sensor and system supply voltage, and some parameters (e.g., the amplifier gain) of the signal conditioning system have been preliminarily selected. Following the above parameters, the sensitivities of the aforementioned measurements can be calculated. These calculated results should be taken as the lower limit of corresponding measurement sensitivity in the above objective functions. 

**Figure 5:**
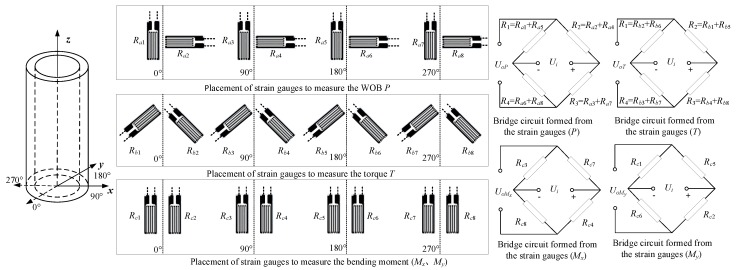
Placement and bridge schemes of strain gauges.

For the current case, this study designs the arrangement scheme of strain gauges on the basis of the relevant references [34,35], as shown in Figure 5. Considering the relatively weak signal of torque measurement, the design optimizes the placement scheme of torque measurement to increase its output voltage. This reduces the corresponding electrical amplification factor to improve measurement stability.

Correspondingly, the output voltage from each measurement bridge can be calculated. Taking the WOB measurement bridge as an example, when the WOB, torque, bending moment, and temperature simultaneously affect the WOB measurement bridge, Equation (11) is established.

(11) $$\{\begin{array}{l} \varepsilon_{R_{a1}}=-\varepsilon_{P}+0+0-\varepsilon_{M_{y}}+\varepsilon_{TP}\\ \varepsilon_{R_{a2}}=\mu \varepsilon_{P}+0+0+\mu \varepsilon_{M_{y}}+\varepsilon_{TP}\\ \varepsilon_{R_{a3}}=-\varepsilon_{P}+0+\varepsilon_{M_{x}}+0+\varepsilon_{TP}\\ \varepsilon_{R_{a4}}=\mu \varepsilon_{P}+0-\mu \varepsilon_{M_{x}}+0+\varepsilon_{TP}\\ \varepsilon_{R_{a5}}=-\varepsilon_{P}+0+0+\varepsilon_{M_{y}}+\varepsilon_{TP}\\ \varepsilon_{R_{a6}}=\mu \varepsilon_{P}+0+0-\mu \varepsilon_{M_{y}}+\varepsilon_{TP}\\ \varepsilon_{R_{a7}}=-\varepsilon_{P}+0-\varepsilon_{M_{x}}+0+\varepsilon_{TP}\\ \varepsilon_{R_{a8}}=\mu \varepsilon_{P}+0+\mu \varepsilon_{M_{x}}+0+\varepsilon_{TP} \end{array},$$

where $\varepsilon_{R_{ai}}$ is the strain of the strain gauge $R_{ai}$ (*i* = 1~8), $\varepsilon_{P}$ is the strain caused by the WOB, $\varepsilon_{M_{x}}$ and $\varepsilon_{M_{y}}$ are the corresponding strains of the bending moment component in the *x* and *y* directions, respectively, $\varepsilon_{TP}$ is the strain value produced by the temperature, and *μ* is the Poisson’s ratio of the material. Thus, the output voltage *U_o__P_* from the WOB measurement bridge can be calculated:

(12) $$U_{oP}=\frac{K_{s}U_{i}}{4}\left|\varepsilon_{R_{1}}-\varepsilon_{R_{2}}+\varepsilon_{R_{3}}-\varepsilon_{R_{4}}\right|=K_{s}U_{i}\left(1+\mu \right)\varepsilon_{P},$$

where *K_s_* is the sensitivity coefficient of the strain gauge and let *K_s_* = 2.1; *U_i_* is the bridge excitation voltage and let *U_i_* = 5 V; $\varepsilon_{R_{i}}$ (*i* = 1~4) is the strain value after the strain gauge is connected in series. For example, according to Figure 5, $\varepsilon_{R_{1}}=\varepsilon_{R_{a1}}+\varepsilon_{R_{a5}}$. Similarly, the output voltage from the measurement bridge of the torque and bending moment can be obtained:

(13) $$U_{oT}=2K_{s}U_{i}\varepsilon_{T},$$

(14) $$U_{oM_{x}}=K_{s}U_{i}\varepsilon_{M_{x}},$$

(15) $$U_{oM_{y}}=K_{s}U_{i}\varepsilon_{M_{y}},$$

where $U_{oT}$*,* $U_{oM_{x}}$, and $U_{oM_{y}}$ are the output voltage from the measurement bridge of the torque and bending moment, respectively. According to the correlation formula of strain and voltage output [26], the calculation formula of the sensitivity boundary of each measurement can be derived as follows.

(16) $$S_{Pb}=\frac{U_{oP}}{\left(1+\mu \right)\cdot U_{i}\cdot K_{s}\cdot P_{\max}}=\frac{U_{fP}/K_{mP}}{\left(1+\mu \right)\cdot U_{i}\cdot K_{s}\cdot P_{\max}},$$

where *S_Pb_* is the sensitivity boundary of the WOB *P*; *P*_max_ is the maximum range value of measurement *P*, and *P*_max_ = 300 kN; *U_fP_* is the maximum voltage output of measurement *P* and let *U_fP_* = 5 V; *K_mP_* is the gain of the measurement signal conditioning circuit and let *K_mP_* = 5000; the Poisson’s ratio *μ* of the 42CrMo material is 0.28.

(17) $$S_{Tb}=\frac{U_{oT}}{2U_{i}\cdot K_{s}\cdot T_{\max}}=\frac{U_{fT}/K_{mT}}{2U_{i}\cdot K_{s}\cdot T_{\max}},$$

where *S_Tb_* is the sensitivity boundary of the torque *T*; *T*_max_ is the maximum range value of measurement *T*, and *T*_max_ = 8 kNm; *U_fT_* is the maximum voltage output of measurement *T* and let *U_fT_* = 5 V; *K_mT_* is the gain of the measurement signal conditioning circuit and let *K_mT_* = 5000.

(18) $$S_{M_{x}b}=\frac{U_{oM_{x}}}{U_{i}\cdot K_{s}\cdot M_{x\max}}=\frac{U_{fM_{x}}/K_{mM_{x}}}{U_{i}\cdot K_{s}\cdot M_{x\max}},$$

(19) $$S_{M_{y}b}=\frac{U_{oM_{y}}}{U_{i}\cdot K_{s}\cdot M_{y\max}}=\frac{U_{fM_{y}}/K_{mM_{y}}}{U_{i}\cdot K_{s}\cdot M_{y\max}},$$

where $S_{M_{x}b}$ and $S_{M_{y}b}$ are the sensitivity boundaries of the bending moments *M_x_* and *M_y_*, respectively; *M_x_*_max_ and *M_y_*_max_ are the maximum range values of the corresponding measurements, respectively, and *M_x_*_max_ = *M_y_*_max_ = 10 kNm; $U_{fM_{x}}$ and $U_{fM_{y}}$ are the maximum voltage outputs of the corresponding measurements, respectively; let $U_{fM_{x}}=U_{fM_{y}}=5V$; $K_{mM_{x}}$ and $K_{mM_{y}}$ are the gains of the corresponding measurement signal conditioning circuits, respectively; let $K_{mM_{x}}=K_{mM_{y}}=5000$. Substituting the above preliminarily selected parameters into Equations (16)–(19), the sensitivity boundaries of the corresponding measurements can be obtained:

$$\{\begin{array}{l} S_{Pb}=\frac{5/5000}{1.28\times 5\times 2.1\times 300,000}=2.48\times 10^{-4}\;\mu \varepsilon /N\\ S_{Tb}=\frac{5/5000}{2\times 5\times 2.1\times 8000}=5.95\times 10^{-3}\;\mu \varepsilon /\mathrm{Nm}\\ S_{Mb}\left(S_{M_{x}b},S_{M_{y}b}\right)=\frac{5/5000}{5\times 2.1\times 10,000}=9.52\times 10^{-3}\;\mu \varepsilon /\mathrm{Nm} \end{array}.$$

#### 4.1.3. Determination of Objective Functions and Constraints

Recalling Section 4.1.1, the model to be built has up to six objective functions, which is bound to introduce high computational complexity. Reasonably transforming some of the objective functions into constraints is one way to simplify a multi-objective optimization problem. According to the calculation results in Section 4.1.2, the bending moment sensitivity can be converted into a constraint condition, and the constraint is its sensitivity boundary. Moreover, according to Figure 4, the influences of the parameters *d* and *t* on the WOB sensitivity and the torque sensitivity are consistent. Therefore, it is only necessary to specify the torque sensitivity as the objective function. It can also be seen from Figure 4 that the trends of the three stiffnesses with parameters are consistent. This means that only one of them needs to be selected as the objective function. In this way, there are only two objective functions left in the model to be built, which greatly simplifies the difficulty of solving the problem.

The constraints of the model also include strength requirements and structural size limitations. In terms of strength, as the weak link of the sub, the elastic cell should have a sufficiently high safety factor *f*. Considering some extreme well conditions that may occur, including bit freezing, touching resistance, bit jumping, and other unforeseen conditions, the safety margin should be increased appropriately. The lower limit of the safety factor *f* is tentatively set at 3. Later adjustments can be made based on the strength check results and the specific conditions on site. In addition, the outer protective shell of the strain gauges is related to the parameters *d* and *t*, so its strength should be considered. The load status of the outer protective shell is relatively simple. According to engineering experience, the outer protective shell needs to pay more attention to its sealing integrity problem. This needs to be studied in subsequent work. The lower limit of the safety factor *f_o_* of the outer protection shell is tentatively set at 1.5.

The selection of structural size parameters should also consider engineering or process requirements. For example, the structure should have a large enough internal diameter to ensure the smooth flow of drilling fluid, the wall thickness should not be too small to avoid mechanical processing difficulties, and some arrangement space of strain gauges and wires should be left in the radial direction. The influence of the parameter *L* on the model is single. In principle, a minimum value that satisfies the layout space of strain gauges and wires can be taken, but the boundary effect at both ends of the elastic cell should also be considered. As mentioned previously, the variable *L* is preliminarily set to 160 mm [19]. The value can be verified or adjusted according to the subsequent finite element analysis. Finally, the multi-objective optimization model characterizing the selection problem of elastic cell structural parameters can be described as Equation (20).

(20) $$\{\begin{array}{l} \begin{array}{l} \underset{X\in N}{\max}\left[S_{T}=f_{1}\left(X\right)\right]\\ \underset{X\in N}{\max}\left[K_{T}=f_{2}\left(X\right)\right] \end{array}\}obj\\ \begin{array}{l} S_{Mb}-f_{3}\left(X\right)\leq 0\\ f_{4}\left(X\right)\cdot f-\sigma_{s}\leq 0\\ f_{5}\left(X\right)\cdot f_{o}-\sigma_{s}\leq 0\\ f_{6}\left(X\right)-D\leq 0 \end{array}\}s.t.\\ \begin{array}{l} X={\left[d,t\right]}^{T}\\ N=\left[\left(35,80\right),\left(15,40\right)\right] \end{array}\}V \end{array}.$$

### 4.2. Model Solution Based on NSGA-II

#### 4.2.1. A Brief Description of NSGA-II

An evolutionary algorithm based on NSGA-II is employed to find a Pareto-optimal set for the objective functions in Equation (20). As mentioned by Shi and Reitz, NSGA is currently one of the best evolutionary multi-objective optimization algorithms [36]. The superiority of NSGA-II derives from its fast non-dominated sorting algorithm, crowding distance function, and elitist strategy. In short, with these techniques, NSGA-II has the ability to quickly approximate the optimal frontier of Pareto and provides a uniformly distributed Pareto-optimal set [37].

**Figure 6:**
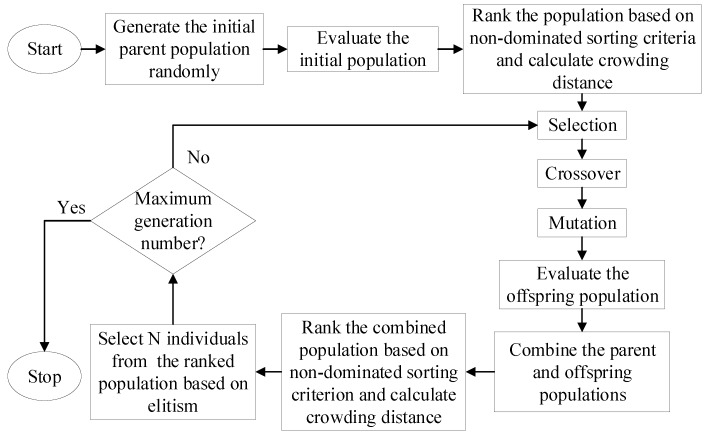
Flowchart of non-dominated sorting genetic algorithm II (NSGA-II).

A brief description of NSGA-II is provided in Figure 6 [38]. First, the initial population is randomly generated and evaluated. Then, the population is sorted using non-dominated sorting criteria and its crowding distance is calculated. Next, the genetic operators of selection, crossover and mutation are applied to generate an offspring population, which is combined with the parent population. After that, the combined population is ranked, and its crowding distance is recomputed. Finally, the best *N* individuals are selected from the combined population based on elitist strategy. The process is repeated until the maximum generation number is reached.

#### 4.2.2. Configuration and Application of NSGA-II

The algorithm needs to be configured before it can be implemented. The first step is the setting of the genetic operators of selection, crossover and mutation. The tournament selection operator with a tournament size of 2 is employed in this study due to its efficiency and simplicity. Arithmetic crossover is a popular crossover operator when a genetic algorithm is applied to optimization. This study adopts the arithmetic crossover operator. For the mutation operator, the classical and common Gaussian mutation operator is adopted.

**Figure 7:**
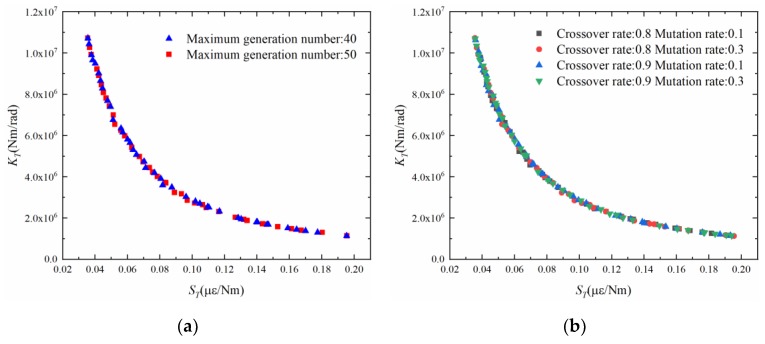
Test results of NSGA-II parameters: (**a**) maximum generation number; (**b**) crossover rate and mutation rate.

Furthermore, the parameters for NSGA-II need to be specified. These parameters include population size, crossover rate, mutation rate and generation number. This study tests the parameters according to the method in reference [39]. The maximum generation number is first tested. This study selected the maximum generation number of 40 and 50 to determine if 40 iterations are enough to approximate the Pareto frontier. The other parameters are set as follows: the population size is 40, the crossover rate is 0.8, and the mutation rate is 0.3. The Pareto frontiers for the 40th and 50th generations are shown in Figure 7a. For these two generation numbers, the corresponding Pareto frontiers are similar. This means that the maximum generation number of 40 can be used for subsequent calculations. Next, several common combinations of the crossover rate and mutation rate are tested. The combination case of these two parameters is shown in Table 2. The corresponding test results are provided in Figure 7b. Uniformly distributed Pareto frontiers can be formed under several common combinations of crossover rate and mutation rate. In other words, the results are not sensitive to these common combinations of crossover rate and mutation rate. Finally, after the aforementioned tests, the following parameters are used in the subsequent analysis: the population size is 40, the maximum generation number is 40, the crossover rate is 0.8, and the mutation rate is 0.3.

In addition to the general configuration, the algorithm has been customized for the problem proposed in this study. It is well known that the maximum Mises stress of a thick-walled cylinder under combined loads (i.e., axial loads, internal and external pressures, bending moment, and torque) is not easily determined theoretically. In this study, the maximum Mises stress of the structure under the above combined loads is searched in a traversal manner by means of the computational efficiency of a computer. The process is combined with the NSGA-II to make the algorithm suitable for the current problem.

**Figure 8:**
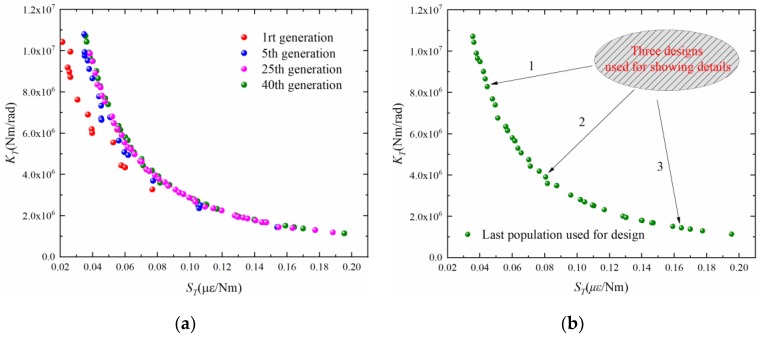
Population evolution process and the last population: (**a**) population evolution process; (**b**) the last population.

After configuration, the algorithm is used to solve the multi-objective optimization problem defined in Equation (20). Figure 8a shows four generations during evolution. As shown in Figure 8a, the Pareto frontier is gradually approximated in the evolution process. Figure 8b shows the last population that will be used for practical optimization. As shown, the current problem has a non-convex Pareto frontier, reflecting the opposition between the two targets. The optimal solution to the problem can be taken from any point on the Pareto curve in Figure 8b.

**Figure 9:**
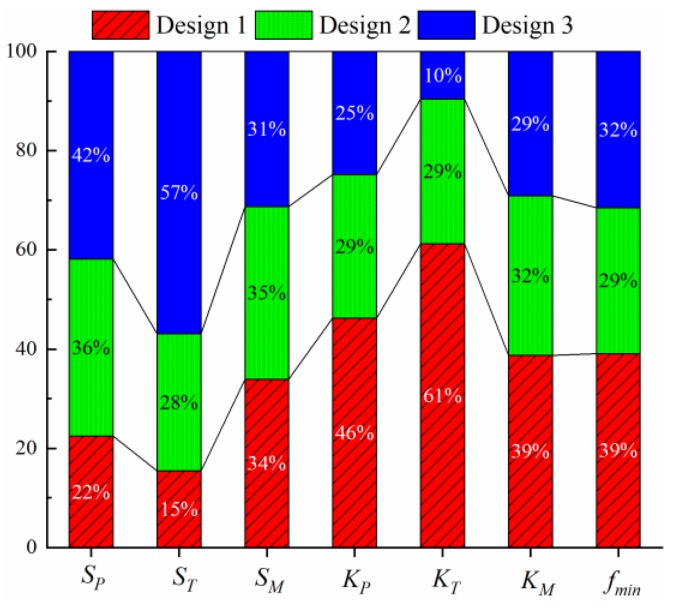
Performance comparison of three designs.

To further demonstrate the results, three solutions on the Pareto frontier are selected with no particular purpose. They are taken from the upper, middle and lower of the Pareto curve, respectively. Table 3 shows these three solutions (i.e., three designs) and their corresponding sensing performance and structural strength indicators. As shown in Table 3, the three designs correspond to different combinations of sensitivity, stiffness and strength. Figure 9 further shows an intuitive comparison of these three designs. It can be seen that this optimization method can provide a flexible solution database. The above analysis is conducted on a computer with Inter(R) Core(TM) i5-8265U CPU and 8 G RAM in MATLAB (R2018a, The MathWorks, Inc., Natick, Massachusetts, USA) environment. The computational time required is only 10 s. This shows the efficiency of the proposed optimization strategy for elastic cell structural parameters.

## 5. Design Verification and Examination Based on FEM

### 5.1. Verification of Measurement Performance and Static Strength

For the size parameters of the elastic cell selected in Section 4.2.2, the relevant verification can be carried out by finite element analysis [40]. In other words, these analyses are used to assess the effectiveness of the multi-objective optimization model. In addition, the strain distribution of the structure can be obtained through finite element analysis, which is infeasible in the above analysis. This study takes design 2 as an example. There are many details in the overall structure of a measurement sub, but the sensing part is mainly studied here, and the other structural features are not considered in the finite element modeling. For the boundary conditions, the lower end surface is set as the fixed end, the upper end is set as the loading position of the WOB, torque, and bending moment, and the inner and outer pressures received by the sub are uniformly applied to the inner and outer walls of the model.

**Figure 10:**
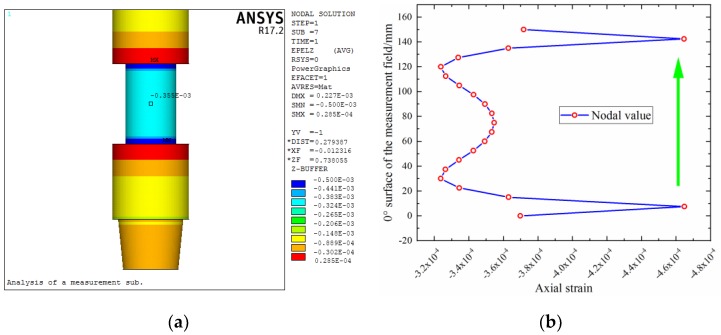
Strain distribution of elastic cell under weight on bit (WOB): (**a**) contour; (**b**) axial distribution.

**Figure 11:**
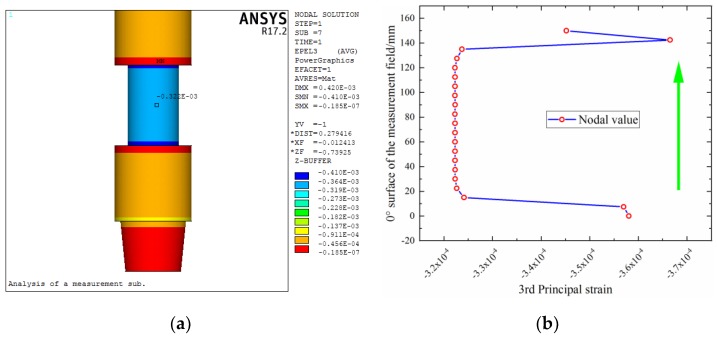
Strain distribution of elastic cell under torque: (**a**) contour; (**b**) axial distribution.

**Figure 12:**
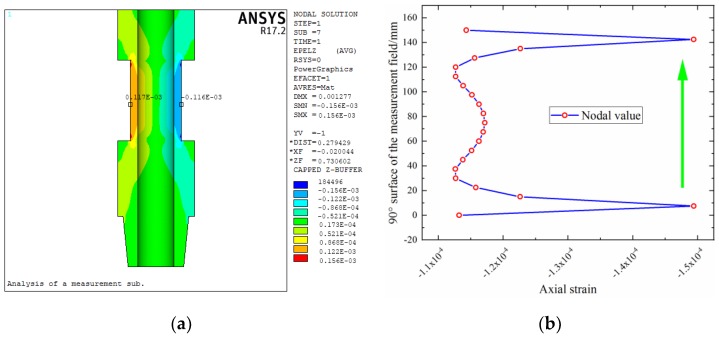
Strain distribution of elastic cell under bending moment: (**a**) contour; (**b**) axial distribution.

The strain of the elastic cell under the action of individual *P*, *T* and *M* is calculated separately, as shown in Figure 10a, Figure 11a and Figure 12a. According to Equations (3), (4), and (6) and Hooke’s law, the strain of the structure under individual *P*, *T* and *M* can be obtained as −3.54 × 10^−4^, −6.41 × 10^−4^ (double), and −1.17 × 10^−4^, respectively. Comparing the finite element results with the theoretical values, the relative error is small, with the maximum value approximately 5%.

It can also be seen from these strain contours that there are strain transition zones at both ends of the elastic cell. Figure 10b, Figure 11b and Figure 12b show the axial distribution of each strain along the measurement area. The maximum value of the length of the stress transition zone at both ends of the measurement area is approximately 30 mm. To eliminate the influence of the boundary effect, it is necessary to avoid this area when pasting strain gauges. The previously selected measurement area with a length of 160 mm can meet such requirements.

**Figure 13:**
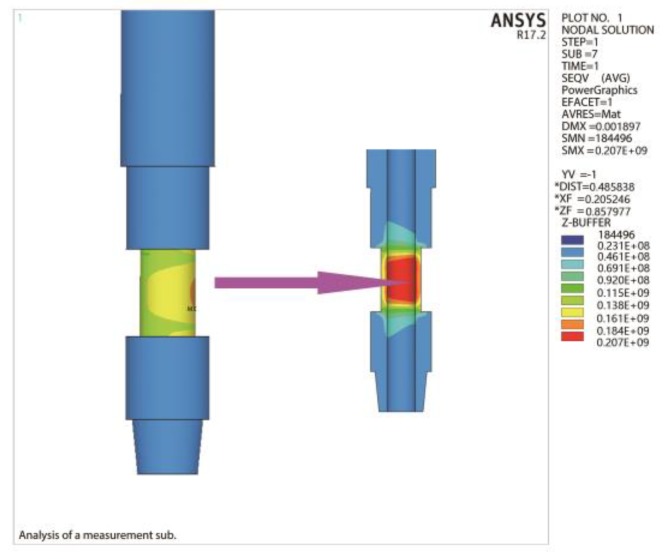
Stress distribution of elastic cell under extreme measurement condition.

To verify the structural strength index of the elastic cell, the finite element analysis is carried out when the measurements above are taken as the maximum range and the sub is subjected to the maximum internal and external pressure. Figure 13 shows the stress contour of the elastic cell under the extreme measurement condition. As shown, the maximum Mises equivalent stress appears on the inner wall of the bending compression side. The maximum Mises stress is 207 MPa, and the relative error is less than 5% compared with the theoretical value. The above static analysis results verify the effectiveness of the multi-objective optimization model.

### 5.2. Transient Dynamic Analysis of Design

When drilling in a complex formation, abnormal drilling phenomena, such as bit bouncing and bit jumping, occur frequently. These phenomena not only will shorten the service life of a bit but also may result in downhole drilling tool fracture. Structural weak points in the drill string are likely to be damaged if they experience severe impact vibration [41]. Therefore, to make the entire design more rigorous, it is necessary to further examine the dynamic response of the elastic cell under impact loads. In this work, two typical extreme conditions are considered, and corresponding transient dynamics finite element models are established.

Bit jumping occurs frequently when drilling with a roller bit. At this time, in addition to the static WOB, the load acting on the drill bit (or rock) also has an impact dynamic load. This dynamic load is represented by the rapid fluctuation of the weight indicator on the drill floor. Obviously, bit jumping makes the drill string bear additional loads, which may endanger the structural integrity of the elastic cell in the measuring sub. For this extreme working condition, the impact dynamic response of the measurement sub is carried out.

When modeling, the analysis can be divided into two stages. (1) The annulus pressure, the pressure inside the sub, the bending moment and the driving torque are applied on the measurement sub, and the prestress status of the structure is calculated. This process is a static analysis. (2) The impact dynamic load caused by bit jumping is applied on the above prestressed model, which is a transient dynamics analysis. The above analysis process can be realized by the “implicit-explicit” sequential solution of the software ANSYS/LS-DYNA [42]. This solution can not only effectively overcome the shortcomings of explicit algorithm in dealing with static problems, but also make full use of its advantages in conducting transient dynamic analysis.

**Figure 14:**
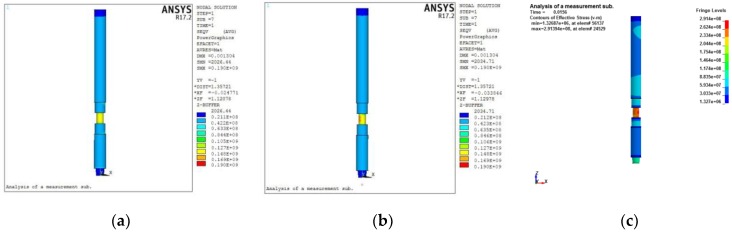
Stage results of the implicit-to-explicit sequential solution: (**a**) implicit solution result; (**b**) initialization model for explicit analysis; (**c**) explicit analysis result.

According to the field measured data, the peak value of the near-bit WOB may reach 3.5 times the average WOB [27,43]. Thus, the impact load applied to this model is set to be 3 times the maximum WOB. The action time of this load is extremely short and is set as 0.03 s [44,45]. Further, this impact dynamic load is simplified as a triangular load-time function. Figure 14 shows the results of the three phases of the entire solution process. Figure 14a shows the result of the implicit analysis. Next, the node result of the implicit solution needs to be written into the dynamic relaxation file (.drelax). The .drelax file is then used to initialize the geometry model for explicit analysis. The initialized model can be regarded as the starting point for explicit analysis. Figure 14b shows the model after initialization. The stress contour shows a small difference from the static analysis due to the element type, but the numerical results are consistent. Figure 14c shows the model equivalent stress contour (0.15 s) obtained by the explicit solution.

**Figure 15:**
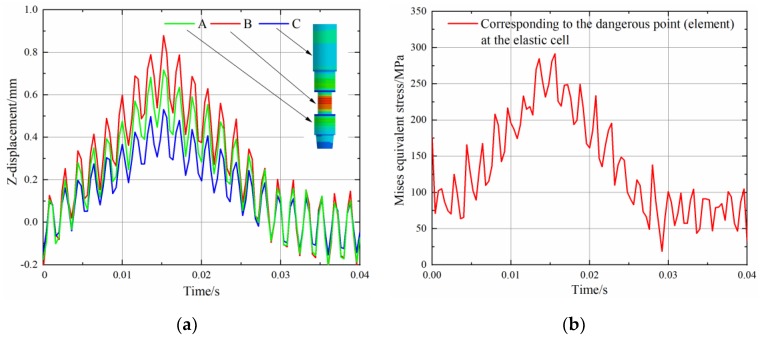
Time-history response of the structure under impact load: (**a**) displacement (**b**) maximum of Mises stress.

To analyze the response characteristics of the sub under impact load, several time-history results are investigated. Figure 15 shows the displacement and the maximum Mises time-history curve of the structure. The overall trend of the curve is consistent with the load form. However, due to the high level of prestress in the structure, a certain oscillation occurs. The *Z*-direction (axial direction) displacement responses of the elements taken from three locations on the sub under impact load are shown in Figure 15a. The element B taken from the elastic cell part has a relatively large displacement. However, no significant displacement saltation occurred. This is mainly due to the stiffness assurance in the design. Figure 15b shows the time-history curve of the maximum Mises stress of the structure. The maximum value appears at the elastic cell part and reaches 291 MPa. This value is increased by approximately 40% compared to the extreme measurement condition. In principle, the current design can withstand the impact under this condition. However, the impact stress has a high sensitivity to defects. For structures with defects, cracks are easily generated when subjected to external shocks [46]. This means that when selecting the material for the design, the material should be inspected for defects. It should be noted that the above-mentioned dynamic characteristics of the structure are not available in a static analysis.

Bit bouncing is also an abnormal drilling phenomenon that occurs during the drilling of a complex formation. This phenomenon is represented by the instantaneous increase of the rotary torque indicator. At this moment, the torsional moment of the drill string is doubled. If the formation drilled contains small caves or fissures, continuous drilling breaks may occur frequently. At this point, the drill string may be subjected to a torque shock. This working condition may jeopardize the structural integrity of the elastic cell in the measurement sub. It is necessary to perform transient dynamic analysis of the measurement sub for this extreme condition.

**Figure 16:**
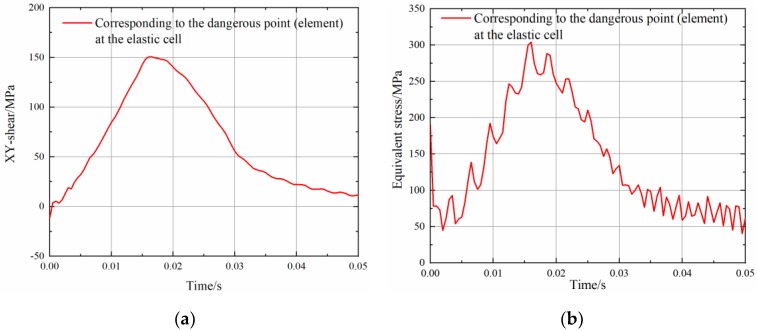
Stress time-history response of sensing area under impact load: (**a**) torsional shear stress (**b**) Mises stress.

The shock response analysis of the sub under this condition is similar to the previous analysis. The boundary conditions are treated similarly when the transient dynamic analysis model is established. Field measured data shows that the downhole torque varies from approximately 20% to 40% of the average torque, while its peak value can reach 2.4 times of the average torque [27,43]. The impact torque applied to the model here is set to be 2 times the maximum torque. Figure 16 shows the stress time-history curve of a dangerous point (element) in the elastic cell. The torsional shear stress of the cross section of the elastic cell increases sharply under impact torque, reflecting the load characteristics of the structure as shown in Figure 16a. This information arouses the interest in further investigating the strength of the structure. Correspondingly, the Mises stress time-history curve for the elastic cell hazardous element is shown in Figure 16b. The peak stress at this position reaches 304 MPa. The proposed structure can withstand the impact under this condition. As discussed previously, the material used in the design should be inspected for defects when such shocks occur frequently.

Although the current design can meet the strength requirements of the above two typical conditions, the significance of dynamic analysis should not be ignored. The main reason is that the service environment of a measurement sub is usually dynamic. Therefore, dynamic analysis is necessary to examine the structural integrity of a design. More specifically, more dynamic working conditions can be analyzed in a specific application. It is likely that there is a case that makes the current design invalid. For this case, the previous optimization model (e.g., the safety factor) can be adjusted based on the corresponding analysis results. This adjustment may be repeated until a better design is obtained. In conclusion, dynamic analysis is a potential means to improve designs and should be incorporated into the design framework of an elastic cell.

## 6. Conclusions

In this paper, a systematic strategy for optimizing the structural parameters of an elastic cell in a near-bit drilling engineering parameters measurement sub is proposed. The focus of this study is a detailed discussion of the developed methodology.

1. The multiple relations among measurement sensitivities, structural stiffnesses and strength in the structural parameters design of an elastic cell were systematically summarized. As *d* and *t* increase, the tension and compression stiffness, torsional stiffness, and bending stiffness of the structure increase, the measurement sensitivities of WOB and torque decrease, and the measurement sensitivity trend of bending moment is not monotonic. Moreover, the relationships among *d*, *t* and the structural strength of an elastic cell are depicted quantitatively.
2. A multi-objective optimization model was established to characterize the multiple relations among measurement sensitivities, structural stiffnesses, and strength in the design of structural parameters. NSGA-II was applied to solve the multi-objective optimization problem. The results showed that the optimization strategy could obtain the optimal solution set of the problem in a short time. This optimization result provided a flexible database for the design of the structural parameters of an elastic cell.
3. The measurement performance and structural strength of the optimization results were verified based on the FEM. The maximum relative error between the simulation results and the theoretical values of the strain in the measurement area is within 5%, which verified the measurement reliability of the structure. For the maximum Mises stress of the elastic cell under the extreme measurement condition, the relative error between the simulated value and the theoretical value is also within 5%. This further validates the strength reliability of the structure. These two results indicate the effectiveness of the proposed optimization strategy.
4. It was proposed that transient dynamics analysis should be used to investigate the dynamic strength of the designed structure to improve the design. The transient dynamic models of the sub under two typical working conditions were established, and the dynamic response of the structure under the corresponding impact load was investigated. The current design could meet the strength requirements of these two working conditions. Nevertheless, the potential of dynamic analysis to provide design feedback should not be overlooked. Dynamic analysis should be incorporated into the entire design process.

## Figures and Tables

**Table 1:** Expressions of basic characters of the elastic cell.

Loads	Sensitivities	Stiffnesses
WOB, *P*	$S_{P}=\frac{4}{E\pi \left[{\left(d+2t\right)}^{2}-d^{2}\right]}$	$K_{P}=\frac{E\pi \left[{\left(d+2t\right)}^{2}-d^{2}\right]}{4L}$
Torque, *T*	$S_{T}=\frac{16\left(d+2t\right)}{\pi G\left[{\left(d+2t\right)}^{4}-d^{4}\right]}$	$K_{T}=\frac{\pi G\left[{\left(d+2t\right)}^{4}-d^{4}\right]}{32L}$
Bending moment, *M*	$S_{M}=\frac{32\left(d+2t\right)}{\pi E\left[{\left(d+2t\right)}^{4}-d^{4}+{\left(d_{o}+2t_{o}\right)}^{4}-d_{o}{}^{4}\right]}$	$K_{M}=\frac{\pi E\left[{\left(d+2t\right)}^{4}-d^{4}+{\left(d_{o}+2t_{o}\right)}^{4}-d_{o}{}^{4}\right]}{64L}$

Note: *S_P_*, *S_T_*, and *S_M_* are the WOB sensitivity, torque sensitivity, and bending moment sensitivity, respectively. *K_P_*, *K_T_*, and *K_M_* are the tension and compression stiffness, torsional stiffness, and bending stiffness, respectively. *E* and *G* are the elastic modulus and shear modulus of the material, respectively. *I* and *J* are the axis moment of inertia and pole moment of inertia of the structure, respectively. *L*, *A*, *d*, and *t* are the length, cross-sectional area, inner diameter, and wall thickness of the elastic cell, respectively. *d_o_* and *t_o_* are the inner diameter and wall thickness of the outer protective shell of strain gauges, respectively.

**Table 2:** Several common combinations of crossover rate and mutation rate.

Combination No.	Crossover Rate	Mutation Rate
Combination 1	0.8	0.1
Combination 2	0.8	0.3
Combination 3	0.9	0.1
Combination 4	0.9	0.3

**Table 3:** Results presentation of three designs.

Designs	Structural Parameters			Sensitivities			Stiffnesses			Strength Indices	
	*d* mm	*t* mm	*L* mm	*S_P_*/10^−3^ με/N	*S_T_*/10^−2^ με/Nm	*S_M_*/10^−2^ με/Nm	*K_P_*/10^9^ N/rad	*K_T_*/10^6^ Nm/rad	*K_M_*/10^7^ Nm/rad	*σ*_emax_/MPa	*f*_min_
1	77	21	160	0.74	4.49	1.14	8.44	8.28	3.25	164	5.68
	152	10	-	-	-	-	-	-	-	336	2.77
2	71	15	160	1.18	8.02	1.17	5.29	3.94	2.69	218	4.27
	152	10	-	-	-	-	-	-	-	340	2.74
3	48	17	160	1.38	16.53	1.05	4.53	1.30	2.44	203	4.58
	152	10	-	-	-	-	-	-	-	342	2.72

Note: The yield strength of 42CrMo is 930 MPa.
